# Age distribution and risk factors for Barrett's esophagus by sex at health check-up settings in Japan

**DOI:** 10.1007/s00535-025-02222-2

**Published:** 2025-02-10

**Authors:** Sho Fukuda, Kenta Watanabe, Dai Kubota, Nobutake Yamamichi, Yu Takahashi, Yoshitaka Watanabe, Kyoichi Adachi, Norihisa Ishimura, Tomoyuki Koike, Hideyuki Sugawara, Kiyotaka Asanuma, Yasuhiko Abe, Takashi Kon, Eikichi Ihara, Kazuhiro Haraguchi, Yoshihiro Otsuka, Rie Yoshimura, Yugo Iwaya, Takuma Okamura, Noriaki Manabe, Akira Horiuchi, Mio Matsumoto, Kengo Onochi, So Takahashi, Tatsuki Yoshida, Yosuke Shimodaira, Katsunori Iijima

**Affiliations:** 1 Department of Gastroenterology, Akita University Graduate School of Medicine, 1-1-1 Hondo, Akita, Akita 010-8543 Japan; 2 Next-Generation Endoscopic Computer Vision, Graduate School of Medicine, The University of Tokyo, Tokyo, Japan; 3 Center for Epidemiology and Preventive Medicine, The University of Tokyo Hospital, Tokyo, Japan; 4 Department of Gastroenterology, Graduate School of Medicine, The University of Tokyo, Tokyo, Japan; 5 Department of Gastroenterology, Kameda Medical Center Makuhari, Chiba, Chiba Japan; 6 Health Center, Shimane Environment and Health Public Corporation, Matsue, Shimane Japan; 7 Second Department of Internal Medicine, Shimane University Faculty of Medicine, Izumo, Shimane Japan; 8 Division of Gastroenterology, Tohoku University Graduate School of Medicine, Sendai, Miyagi Japan; 9 Cancer Detection Center, Miyagi Cancer Society, Sendai, Miyagi Japan; 10 Division of Endoscopy, Yamagata University Hospital, Yamagata, Yamagata Japan; 11 Department of Gastroenterology, Yamagata Saisei Hospital, Yamagata, Yamagata Japan; 12 Department of Medicine and Bioregulatory Science, Graduate School of Medical Sciences, Kyushu University, Fukuoka, Fukuoka Japan; 13 Department of Gastroenterology, Harasanshin Hospital, Fukuoka, Fukuoka Japan; 14 Medical Treatment Corporate Foundation Group Hakuaikai Medical Checkup Center Wellness, Fukuoka, Fukuoka Japan; 15 Department of Gastroenterology, Shinshu University School of Medicine, Matsumoto, Nagano Japan; 16 Department of Gastroenterology, Aizawa Hospital, Matsumoto, Nagano Japan; 17 Division of Endoscopy and Ultrasonography, Department of Clinical Pathology and Laboratory Medicine, Kawasaki Medical School, Kurashiki, Okayama Japan; 18 Digestive Disease Center, Showa Inan General Hospital, Komagane, Nagano, Japan; 19 Department of Gastroenterology, Sapporo Medical Center, NTT EC, Sapporo, Hokkaido Japan; 20 Department of Gastroenterology, Sapporo Cancer Screening Center, Public Interest Foundation Hokkaido Cancer Society, Sapporo, Hokkaido Japan; 21 Department of Gastroenterology, Omagari Kosei Medical Center, Omagari, Akita Japan; 22 Department of Gastroenterology, Yuri Kumiai General Hospital, Yurihonjo, Akita Japan

**Keywords:** Barrett’s esophagus, Sex, Age, Body mass index, Waist circumference

## Abstract

**Background:**

Given the high prevalence of esophageal adenocarcinoma and Barrett's esophagus (BE), a precancerous lesion, among males, it is important to understand the characteristics of BE by sex to develop an effective endoscopic surveillance program in Japan. The present study examined the age distribution and risk factors for BE in the Japanese health check-up cohort by sex.

**Methods:**

The data set at baseline of our preceding multicenter study, comprising a total of 33,478 individuals who underwent upper endoscopic screening at 17 health check-up institutes across Japan, was utilized. BE and long-segment BE (LSBE) were defined as a columnar-lined esophagus ≥ 1 cm and ≥ 3 cm, respectively. Logistic regression analyses were performed to ascertain the factors associated with BE.

**Results:**

BE was relatively common (10–20%) across all 10-year age groups in men and women. Although the prevalence of LSBE was rare (0.2%), it began to increase at younger ages in men. In the multivariable analysis of the male cohort, while body mass index (BMI) was negatively associated with BE with an adjusted odds ratio (95% confidence interval) of 0.84 (0.74–0.95), waist circumference (WC) was positively associated with 1.26 (1.13–1.41). Furthermore, this association was more pronounced in LSBE. In contrast, no such association was observed between BMI or WC and BE in women.

**Conclusion:**

The nationwide Japanese multicenter study yielded insights into the age distribution of BE in the Japanese population. Furthermore, our findings indicate that a low BMI/high WC represents a significant risk factor for BE in the Japanese male population.

**Supplementary Information:**

The online version contains supplementary material available at 10.1007/s00535-025-02222-2.

## Introduction

Several decades behind the situation in Europe and the United States, esophageal adenocarcinoma (EAC) began to increase in Japan around 2010 [1, 2], and it is necessary to take measures to deal with Barrett's esophagus (BE), its precancerous lesion [3, 4]. In Japan, where gastric cancer is common, endoscopic screening for gastric cancer is currently recommended for all men and women over 50 years of age, and BE and EAC could be detected at that time [5]. Nevertheless, in light of the decline in the incidence of gastric cancer resulting from a reduction in the prevalence of *H. pylori* infection [6], the implementation of screening strategies based on risk stratification for gastric cancer is being contemplated, rather than a universal screening approach based on age [5]. Under such circumstances, it is necessary to introduce a unique screening system for BE and EAC in Japan.

A major characteristic of EAC is the large sex difference, with males 5–10 times more common than females [7]. This male predominance is already present at the stage of BE (2–3 times) [7]. Therefore, it should be useful to establish different criteria for men and women in surveillance for BE and EAC. Since sex and age are the most basic and publicly available patient information, knowing the distribution of BE by these factors should be highly beneficial as a preliminary step in developing an effective surveillance system. Furthermore, the identification of risk factors for BE by sex may prove useful in further narrowing down high-risk groups.

A recent retrospective cohort study of over 30,000 participants at 17 health screening institutes throughout Japan has enabled us to determine the incidence of EAC in the Japanese population by each segment of BE [8]. In that study, 90% of the subjects diagnosed with EAC were male, thereby reproducing the strong male predominance observed in the Japanese population [8]. In the present study, we examined the age distribution and risk factors for BE in the Japanese health check-up cohort by sex, using data registered at baseline in this cohort.

## Methods

All individuals who underwent upper endoscopic screening as part of annual health check-ups at 17 participating institutes during the entry period established at each institution were retrospectively enrolled in the preceding study [8], and the entry period ranged from several months to a whole year between January 2013 and December 2017, depending on the processing capacity of each institute. Participants who had histories of surgery for the upper gastrointestinal (GI) tract (*n* = 332) or poor retrieved endoscopic images at entry (e.g., the absence of clear images of the gastro-esophageal junction, *n* = 20) were excluded from the study. In the present study, we utilized the baseline clinical data at entry, including the diagnosis of BE. This study was reviewed and approved by the ethics committee of Akita University Hospital (2946) and each participating hospital.

### Data collection

The clinical data set included information on the patient's sex, age, and drinking and smoking habits, as self-reported by the patients during the health check-up. Drinking and smoking statuses were dichotomized into two categories: never and ever. Additionally, data on body mass index (BMI) and waist circumference (WC) were collected based on physical measurements during the health check-up. The criteria for defining central obesity were based on the Japanese metabolic syndrome criteria, with a WC of ≥ 85 cm for men and ≥ 90 cm for women considered to indicate the presence of central obesity [9]. In addition to the BE diagnosis described below, endoscopic gastric atrophy was evaluated and classified according to the classification of Kimura and Takemoto into three groups: absent, mild to moderate (closed-type), or extensive (open-type) [10]. Furthermore, reflux esophagitis (RE) more than grade A in the Los Angeles classification was also endoscopically diagnosed [8].

### BE diagnosis

As previously stated, following the consensus meeting to diagnose BE, presence or absence of BE and, if present, its length were retrospectively assessed at each institute using the retrieved endoscopic images recorded at entry [8]. The gastro-esophageal junction was defined as the oral side end of the fold continuous with the gastric lumen or the anal side end of the palisade vessel [11]. BE was diagnosed as columnar-lined epithelium extending from the junction to the esophagus in a continuous manner in the distal esophagus of any length at endoscopy according to the criteria of the Japanese Esophageal Society [12]. Subsequently, the participants were then divided into four groups according to the presence or absence of BE and, if applicable, the maximum length of the BE segment: absent, columnar-lined epithelium < 1 cm (USSBE), 1 and < 3 cm (SSBE), and ≥ 3 cm (LSBE) [8]. In the present study, BE was defined as a columnar-lined esophagus ≥ 1 cm (SSBE plus LSBE) due to the poor inter-observer agreement observed for USSBE [8], which could lead to significant misclassification of absent BE. Subsequently, to identify the unique characteristics of BE, subjects with USSBE were excluded from the analysis for risk factors for BE. The length of BE was estimated using retrieved endoscopic images of the esophago-gastric junction independently at each institute following the consensus meeting.

### Statistics

Continuous variables were expressed as medians with interquartile ranges and were compared using the Mann–Whitney *U* test. Categorical variables were expressed as frequencies and proportions and compared using Fisher's exact test or chi-square test as appropriate. Both continuous and categorical variables were assessed for BMI and WC, employing the appropriate cutoffs (e.g., 25 kg/m^2^ for BMI and 85 cm for men and 90 cm for women for WC). The prevalence of BE was presented separately for men and women in the 10-year age group between the ages of 30 and 70 with a sufficient number of participating subjects (e.g., more than 500 cases). Logistic regression analyses were conducted to investigate the factors associated with BE, and the results were expressed as odds ratios (ORs) and 95% confidence intervals (CIs). All statistical analyses were conducted with the EZR software program (Saitama Medical Center, Jichi Medical University, Saitama, Japan) [13], and *P* values of < 0.05 were considered statistically significant.

## Results

**Fig. 1:**
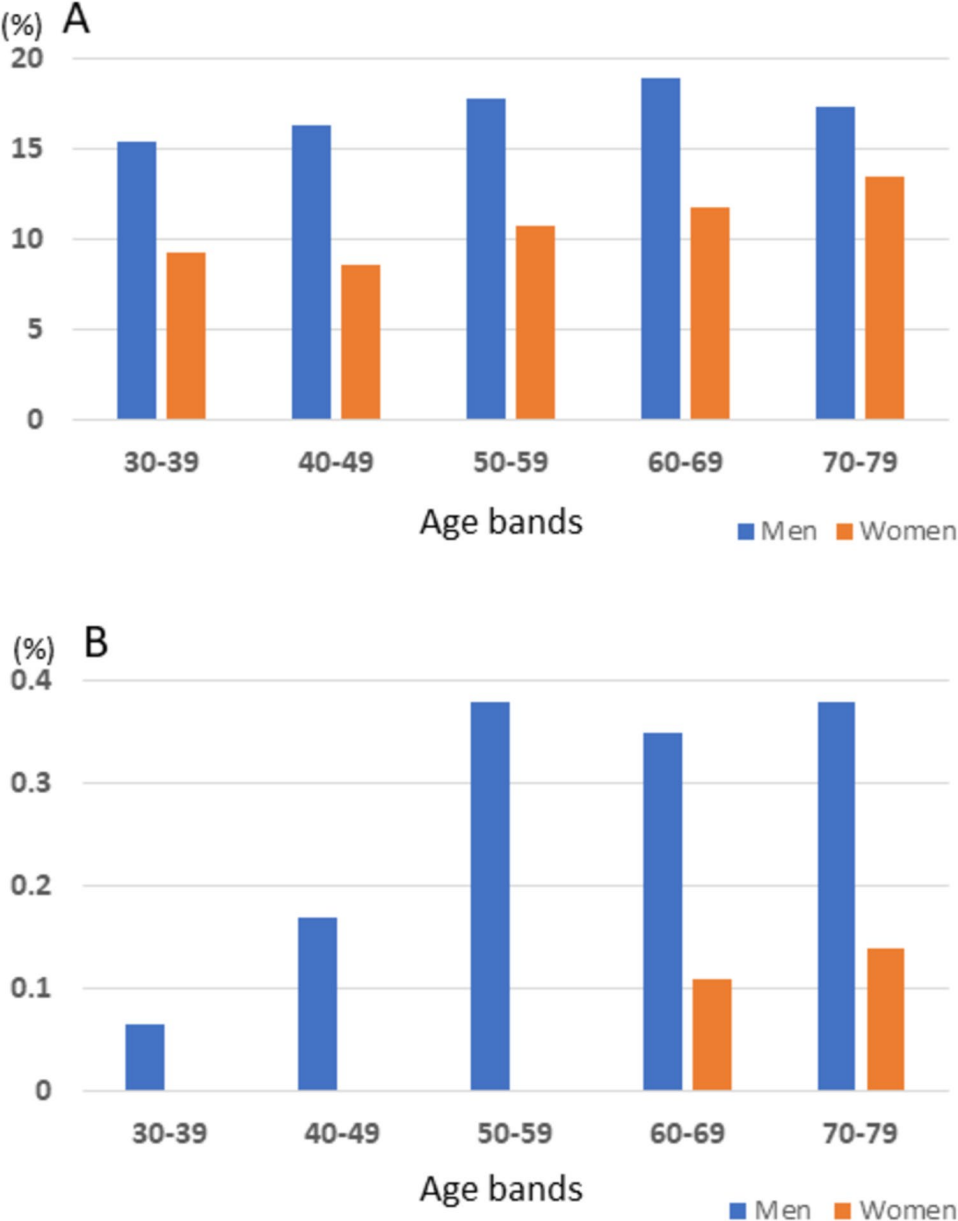
The prevalence of Barrett’s esophagus defined by ≥ 1 cm (**A**) or long-segment Barrett’s esophagus defined by ≥ 3 cm (**B**) in each 10-year age group by sex

Overall, a total 33,478 (20,411 men and 13,067 women) participants were subjects for BE prevalence. Figure 1 shows the prevalence of BE defined by ≥ 1 cm (A) or LSBE (B) in each 10-year age group by sex. BE is a relatively common diagnosis in both men and women in the 30–70 s age range although it is consistently more prevalent in men across all age groups. The prevalence of BE is relatively uniform, affecting 15–20% of men and demonstrating a slight upward trajectory from 8 to 14% with age in women (Fig. 1A). In contrast, in addition to the rarity of LSBE, LSBE demonstrated a distinct age- and sex-specific distribution pattern. In men, the prevalence of LSBE begins to rise in their 30 s and reaches a plateau of 0.3–0.4% in their 50 s. In contrast, in women, it is not observed until their 50 s, with a slight increase to 0.1–0.2% in women aged 60 and above (Fig. 1B).

After excluding USSBE subjects, Table 1 presents a comparison of the investigated factors between the BE or LSBE and the control group. The proportion of males in the BE group was 72.5%, which was significantly higher than that observed in the control group (54.4%, *P* < 0.001). Moreover, in the LSBE group, the proportion of males reached 93.8%. The majority of the other investigated factors exhibited statistically significant differences between the BE or LSBE groups and the control group.

**Table 1:** Comparisons of the investigated factors between the group of Barrett’s esophagus (or long-segment Barrett’s esophagus) and the control group

Variables	Control (*n* = 17,884)	BE (*n* = 4953)	LSBE (*n* = 64)	*P* value (cont. vs. BE)	*P* value (cont. vs. LSBE)
Age (years)	53 (46, 60)	55 (47, 62)	58 (51, 62)	< 0.001	< 0.001
Age ≥ 65 years (*n*, %)	2502 (14)	833 (16.8)	8 (12.5)	< 0.001	0.478
Sex (male, %)	9656 (54.0)	3590 (72.5)	60 (93.8)	< 0.001	< 0.001
BMI (kg/m^2^)	22.6 (20.6, 24.8)	23.1 (21.2, 25.2)	24.1 (22.4, 25.7)	< 0.001	0.002
BMI ≥ 25 kg/cm^2^ (*n*, %)	3745 (23.6)	1204 (26.9)	14(21.9)	< 0.001	0.64
Waist circumference (cm)	81.0 (75.0, 87.5)	83.5 (77.8, 89.0)	86.5 (82.8, 90.0)	< 0.001	< 0.001
Central obesity (*n*, %)^a^	4053 (28.9)	1550 (38.2)	27(42.2)	< 0.001	0.025
Ever Smokers (*n*, %)	6319 (45.5)	1866 (46.3)	27 (64.3)	0.369	0.002
Ever Drinkers (*n*, %)	10,663 (75.7)	3023 (74.1)	40 (95.2)	0.066	0.019
Gastric mucosal Atrophy: None / closed / open (*n*, %)	10,035 (56.1) / 4236 (23.7) / 3603 (20.1)	2496 (50.4) / 1253 (25.3) / 1203 (24.3)	51 (80) / 9 (14) / 4 (6) / 0 (0)	< 0.001	< 0.001
Reflux esophagitis (*n*, %)	1268 (8.4)	1106 (23.3)	20 (31.7)	< 0.001	< 0.001

*BE* Barrette’s esophagus, *BMI* body mass index, *LSBE* long-segment Barrett’s esophagus

^a^Central obesity is defined as a waist circumference ≥ 85 cm for men and ≥ 90 cm for women

Male sex is consistently associated with BE with an OR (95% CI) 2.2 (2.1–2.4), *P* < 0.001 in the univariable analysis, 2.4 (2.2–2.6), *P* < 0.001 in both multivariable analyses. In the univariable analysis, both obesity-related factors (BMI and WC) were found to be positively associated with BE, whether as a continuous variable or a dichotomous variable. Nonetheless, in multivariable models in which the two factors were incorporated together, they demonstrated a paradoxical relationship with BE. On the one hand, there is a significant negative association between BMI as a continuous variable and BE, with an adjusted OR (95% CI) 0.93 (0.91–0.94), *P* < 0.001. This significance persisted even when BMI was treated as a dichotomous variable, with an adjusted OR (95% CI) 0.89 (0.80–0.99), *P* = 0.04. Conversely, WC is consistently and positively associated with BE, with an adjusted OR (95% CI) of 1.03: (1.02–1.04) as a continuous variable and 1.23 (1.11–1.37) as a dichotomous variable, both with a *P* value less than 0.001 (Table 2).

**Table 2:** Regression analyses for factors associated with Barrett’s esophagus in the entire cohort

Variables	Ref	Univariable		Multivariable 1		Multivariable 2	
		OR (95% CI)	*P* value	AOR (95% CI)	*P* value	AOR (95% CI)	*P* value
Age ≥ 65 years	< 65	1.24 (1.14–1.35)	< 0.001	1.01 (0.90–1.14)	0.83	1.04 (0.92–1.17)	0.54
Male Sex	Female	2.24 (2.09–2.40)	< 0.001	2.44 (2.22–2.67)	< 0.001	2.43 (2.21–2.66)	< 0.001
BMI	Continuous	1.04 (1.03–1.05)	< 0.001	0.93 (0.91–0.94)	< 0.001		
≥ 25 kg/m^2^	< 25	1.19 (1.10–1.28)	< 0.001			0.89 (0.80–0.99)	0.04
Waist circumference	Continuous	1.02 (1.02–1.02)	< 0.001	1.03 (1.02–1.04)	< 0.001		
Central obesity^a^ (Yes)	No	1.52 (1.41–1.63)	< 0.001			1.23 (1.11–1.37)	< 0.001
Ever smokers	Never	1.03 (0.96–1.11)	0.36	0.81 (0.75–0.88)	< 0.001	0.81 (0.75–0.88)	< 0.001
Ever drinkers	Never	0.93 (0.86–1.00)	0.06	0.70 (0.63–0.76)	< 0.001	0.69 (0.63–0.76)	< 0.001
Gastric atrophy							
Closed-type	None	1.19 (1.10–1.28)	< 0.001	1.04 (0.96–1.14)	0.35	1.05 (0.96–1.15)	0.28
Open-type	None	1.34 (1.24–1.45)	< 0.001	1.00 (0.91–1.11)	0.96	1.01 (0.92–1.12)	0.82

BMI and waist circumference were entered as continuous variables in multivariable analysis 1, and as categorical variables in multivariable analysis 2

*AOR* adjusted odds ratio, *BMI* body mass index, *CI* confidence interval, *OR* odds ratio

^a^Central obesity is defined as a waist circumference ≥ 85 cm for men and ≥ 90 cm for women

Upon repeating the same analysis on a male and female basis, it was observed that the emerging paradoxical association between BMI or WC and BE was confined to the male cohort (Table 3). In the male cohort, BMI ≥ 25 kg/m^2^ was significantly negatively associated with BE in multivariable analysis with an OR (95% CI) 0.84 (0.74–0.95), *P* = 0.004, while central obesity defined as WC ≥ 85 cm was significantly positively associated with BE in multivariable analysis with an adjusted OR (95% CI) 1.26 (1.13–1.41), *P* < 0.001. In contrast, no such significant association was observed in the female cohort: adjusted ORs (95% CI) 1.12 (0.89–1.40), *P* = 0.34 for BMI ≥ 25 kg/m^2^ and 1.10 (0.85–1.43), *P* = 0.47 for central obesity, defined as WC ≥ 90 cm in multivariable analysis. In the analysis restricted to LSBEs in the male cohort (Supplemental Table 1), the paradoxical association was more pronounced in the multivariable analysis with OR (95% CI) 0.48 (0.22–1.04), *P* = 0.06 for BMI ≥ 25 kg/m^2^ and 3.18 (1.54–6.53) for central obesity, *P* = 0.002 although the former did not reach statistical significance due to insufficient number of subjects. The small number of female LSBEs (*n* = 4) precluded the same analysis in the female cohort. Meanwhile, endoscopic gastric atrophy, especially open-type one, showed a strong negative association with LSBE in the male cohort.

**Table 3:** Regression analyses for factors associated with Barrett’s esophagus separately in men and women

Variables	Ref	Men				Women			
		Univariable		Multivariable		Univariable		Multivariable	
		OR (95% CI)	*P* value	AOR (95% CI)	*P* value	OR (95% CI)	*P* value	AOR (95% CI)	*P* value
Age ≥ 65 years	< 65	1.08 (0.97–1.20)	0.15	0.96 (0.84–1.10)	0.56	1.49 (1.28–1.75)	< 0.001	1.28 (1.03–1.60)	0.026
BMI ≥ 25 kg/m^2^	< 25	0.98 (0.89– 1.07)	0.61	0.84 (0.74–0.95)	0.004	1.23 (1.05–1.45)	0.009	1.12 (0.89–1.40)	0.34
Central obesity^a^ (Yes)	No	1.16 (1.06–1.26)	< 0.001	1.26 (1.13–1.41)	< 0.001	1.27 (1.05–1.54)	0.012	1.10 (0.85–1.43)	0.47
Ever smokers	Never	1.03 (0.96–1.11)	0.37	0.88 (0.80–0.96)	0.005	0.62 (0.53–0.73)	< 0.001	0.59 (0.49–0.71)	< 0.001
Ever drinkers	Never	0.93 (0.86–1.00)	0.065	0.64 (0.56–0.72)	< 0.001	0.72 (0.63–0.81)	< 0.001	0.82 (0.71–0.94)	0.004
Gastric atrophy									
Closed-type	None	1.19 (1.10–1.23)	< 0.001	1.05 (0.95–1.17)	0.35	1.20 (1.05–1.38)	0.009	1.06 (0.91–1.24)	0.47
Open-type	None	1.34 (1.24–1.45)	< 0.001	1.07 (0.95–1.20)	0.27	1.24 (1.06–1.45)	0.006	0.88 (0.72–1.07)	0.21

*AOR* adjusted odds ratio, *BMI* body mass index, *CI* confidence interval, *OR* odds ratio

^a^Central obesity is defined as a waist circumference ≥ 85 cm for men and ≥ 90 cm for women

Furthermore, the male subjects were classified into four groups based on appropriate cutoffs for BMI and WC, and the odds ratios of the four obesity-related categories for BE were estimated (see Supplemental Table 2). Nevertheless, the high BMI/low WC category constituted only 1.8% of the total population, and no significant difference was observed in the relative risk for BE between the high BMI/low WC category and the low BMI/low WC category. Consequently, the two aforementioned categories were merged, and the male subjects were divided into three groups: any BMI/low WC, high BMI/high WC, and low BMI/high WC, which accounted for 48.6%, 32.5%, and 18.9% in the targeted population, respectively. The multivariable analysis demonstrated that, when any BMI/low WC category was used as a reference, there was no statistically significant association between the high BMI/high WC category and BE with an OR (95% CI) 1.1 (0.99–1.2), the low BMI/high WC category was found to be consistently associated with BE, with an OR (95% CI) of 1.4 (1.2–1.5), *P* < 0.001. This outcome was particularly evident in the context of LSBE. In other words, while there was no statistically significant association between the high BMI/high WC category and LSBE (OR 1.6, 95% CI 0.73–3.5, *P* = 0.24), a stronger association was demonstrated between the low BMI/high WC category and LSBE (OR: 3.2, 95% CI 1.5–6.7, *P* = 0.002) (Table 4).

**Table 4:** Risks for Barrett’s esophagus (or long-segment Barrett’s esophagus) in men according to the 3 obesity categories

Obesity category	BE vs. control					LSBE vs. control				
	*N* (BE/control)	Univariable		Multivariable^a^		*N* (LSBE/control)	Univariable		Multivariable^a^	
		OR (95% CI)	*P* value	AOR (95% CI)	*P* value		OR (95% CI)	*P* value	AOR (95% CI)	*P* value
Any BMI / Low WC	1421/4083	Ref		Ref		14/4083	Ref		Ref	
High BMI / High WC	836/2127	1.13 (1.02–1.25)	0.017	1.1 (0.99–1.22)	0.07	11/2127	1.51 (0.68–3.33)	0.31	1.61 (073–3.5)	0.24
Low BMI / High WC	657/1400	1.35 (1.21–1.51)	< 0.001	1.37 (1.23–1.54)	< 0.001	15/1400	3.12 (1.5–6.49)	0.02	3.22 (1.55–6.7)	0.002

^a^Adjusted by age, smoking and drinking status, and gastric atrophy

*AOR* adjusted odds ratio, *BE* Barrette’s esophagus, *BMI* body mass index, *CI* confidence interval, *LSBE* long-segment Barrett’s esophagus, *OR* odds ratio, *WC* waist circumference

Finally, as a reference, additional regression analyses were performed to investigate the factors associated with RE in place of BE in the entire cohort (Supplemental Table 3). Interestingly, both BMI ≥ 25 kg/m^2^ and central obesity were positively associated with RE with ORs (95% CI) 1.3 (1.2–1.5), *P* < 0.001 and 1.6 (1.4–1.8), *P* < 0.001, respectively.

## Discussion

The present multicenter study, which encompasses 17 health check-up institutes throughout Japan, has successfully demonstrated the sex-related difference in age distribution and risk factors for BE. In particular, we identified a distinctive, paradoxical correlation between two prevalent obesity-related variables and BE, which was exclusively observed in males. Specifically, our findings revealed that a combination of low BMI and high WC was associated with an increased risk of BE in males.

This multicenter study is the first to determine the prevalence of BE by sex within each Japanese age cohort. The overall prevalence of BE is relatively common in both men and women across all age groups in Japanese health check-up settings although it is consistently more prevalent in men than in women. This distribution pattern appears to differ significantly from that observed in Western countries [14, 15], where endoscopic surveillance for BE was introduced in response to a marked increase in EAC [3]. The discrepancy is likely attributable to disparate international diagnostic criteria for BE (e.g., the necessity for histologic confirmation) or distinct target populations (e.g., health screening vs. subjects presenting with heartburn) [11]. Whether this commonly seen BE in Japan should be included in surveillance depends on its carcinogenic potential [8].

Conversely, LSBE is a rare condition, accounting for 0.2% of the entire cohort. Additionally, there are notable discrepancies in the age distribution between men and women. In men, LSBE starts to appear in the 30 s and reaches a plateau in the 50 s, while in women, it is not seen at all until the 50 s and becomes slightly more common in the 60 s–70 s. Thus, the 20- to 30-year time lag observed in the prevalence of LSBE by sex in Japan is consistent with the findings on the entire BE reported from Europe and the United States [14, 15]. Therefore, LSBE diagnosed in Japan, at least, is considered to refer to the same condition as BE diagnosed in Europe and the United States despite different international diagnostic criteria for BE [11]. LSBE has been confirmed to have a high cancer incidence in Japan [16, 17], comparable to that in Europe and the United States, and should be a target for surveillance. Given that BE is known to rarely extend its length throughout life once it appears [18–20] and that the prevalence of LSBE in Japanese men reaches a plateau in their 50 s, it may be recommended that periodic endoscopic surveillance be initiated in men at the age of 50. In light of a recent study from the United States that demonstrated an increasing prevalence of BE in the young population, which led to the proposal of an adjustment to the age cut-off to 45 years [21], it is imperative to maintain a continuous and meticulous monitoring of the age distribution of BE in Japan. In contrast, women may be an unlikely target for surveillance as EAC is exceedingly rare [8], and LSBE occurs only slightly at an older age.

Although obesity is a well-recognized risk factor for BE in Western countries [3, 4], it is less clear in Asian countries as a previous meta-analysis showed no significant association between BE and simple obesity represented by BMI in Asians [22]. Several studies in the Japanese population investigated the association between BMI and BE with conflicting outcomes, and few dealt with WC as an indicator of central obesity [23–27]. In the present study, when incorporating BMI and WC in an analytical model together, we found paradoxical associations of the two obesity-related variables with BE, namely BMI is negatively associated with BE while central obesity is positively associated with it. Further, such paradoxical association was observed only in men and is more pronounced in LSBE. Notably, this phenomenon has never been identified in reports on BE in Western countries [28, 29]. Very interestingly, the emerging paradoxical association of BMI and WC with BE in the current study, e.g., low BMI/high WC is associated with BE, seems to coincide with a recent finding from Japan that while BMI is negatively associated with early-stage EAC, abdominal obesity measured by visceral fat area on an abdominal CT image is positively associated with it [30]. Thus, low BMI/high WC seems to be a common risk factor for both BE and EAC in Japanese subjects.

BMI and WC are the two most commonly utilized conventional measures for the diagnosis of obesity. However, BMI is unable to identify adipose tissue and thus is unable to discriminate between fat and fat-free lean mass. Accordingly, the emerging paradoxical association may be explained by the new concept of "sarcopenic obesity," in which the co-existence of obesity and sarcopenia could occur with aging [31, 32]. For example, the loss of muscle mass due to sarcopenia results in a reduction in physical activity and the accumulation of visceral fat. The association between sarcopenic obesity and various age-related diseases, including cancer, has recently attracted considerable interest [31–33], and it is noteworthy that Asians are at a greater risk of sarcopenic obesity, including abdominal obesity and low muscle mass, than Caucasians [34, 35]. Therefore, the present study proposes that sarcopenic obesity may be linked to not only EAC but also its precursor BE. This suggests that the identification of candidates for BE surveillance could be further refined by the two easily available obesity-related variables, namely BMI and WC.

Interestingly, RE, regarded as a precursor condition to BE, exhibited a consistent positive association with the two obesity-related variables (See supplemental Table 3). Therefore, the relationship between "obesity" and BE/RE is not uniform. While general obesity is sufficient to increase the risk of developing RE through mechanically induced reflux, sarcopenic obesity is required to increase the risk of BE. Visceral fat, a prominent feature of sarcopenic obesity, is known to be metabolically active and secretes various pro-inflammatory cytokines, such as leptin and adiponectin [4]. These circulating adipokines, released from adipose tissue, may play a crucial role in the pathogenesis of BE [4].

In addition to the obesity-related factors, gastric atrophy is negatively associated with LSBE in this study. Notably, advanced open-type gastric atrophy is identified as a strongly protective factor (e.g., OR = 0.015). This finding is consistent with previous studies in Japan, which have demonstrated that a sufficient amount of gastric acid from a non-atrophic (*H. pylori*-negative) healthy stomach is a prerequisite for BE, particularly LSBE [36]. In Japan, the prevalence of LSBE is expected to increase in future as the rate of *H. pylori* infection in the general population declines.

The strength of this study is that the data were collected from multiple screening facilities throughout Japan and is considered to reflect the actual status of screening in Japan. In addition, to begin this study, a consensus meeting was held regarding the diagnosis of BE to unify the diagnosis. In contrast, a dearth of information regarding certain associated factors represents a potential limitation of this study. Consequently, a multivariable regression analysis was conducted on a subset of subjects, resulting in the exclusion of 20.8% of the original sample. However, since these patients' information are basic data systematically collected at screening facilities, the missing data observed in this study can be attributed to difficulties in providing data at each facility, rather than to individual patient characteristics. Further, due to the absence of a standardized response format for smoking and drinking among the participating institutes, this study was only able to categorize these behaviors as either "ever" or "never." Consequently, the results of this study, which demonstrated a negative correlation between these lifestyle habits and BE, should be interpreted with caution.

In conclusion, this nationwide Japanese multicenter study revealed age distribution and risk factors for BE in the Japanese population, stratified by sex. In particular, we found low BMI/high WC is a strong risk factor for BE in the Japanese male population. The findings will provide valuable information for establishing an efficient surveillance strategy for BE in Japan.

## Supplementary Information

Below is the link to the electronic supplementary material.

Supplementary file1 (DOCX 23 KB)

## Abbreviations

BE: Barrette’s esophagus
BMI: Body mass index
CI: Confidence interval
EAC: Esophageal adenocarcinoma, LSBE, long-segment Barrett’s esophagus
OR: Odds ratio
RE: Reflux esophagitis
SSBE: Short-segment Barrett’s esophagus
USSBE: Ultra-short-segment Barrett’s esophagus
WC: Waist circumference

## References

[1] Koizumi S, Motoyama S, Iijima K. Is the incidence of esophageal adenocarcinoma increasing in Japan? Trends from the data of a hospital-based registration system in Akita Prefecture, Japan. J Gastroenterol. 2018;53:827–33. 29134330 10.1007/s00535-017-1412-4

[2] Matsuno K, Ishihara R, Ohmori M, et al. Time trends in the incidence of esophageal adenocarcinoma, gastric adenocarcinoma, and superficial esophagogastric junction adenocarcinoma. J Gastroenterol. 2019;54:784–91. 30927083 10.1007/s00535-019-01577-7

[3] Spechler SJ, Souza RF. Barrett’s esophagus. N Engl J Med. 2014;371:836–45. 25162890 10.1056/NEJMra1314704

[4] Iijima K. Etiologic factors for Barrett’s esophagus: toward countermeasures in Asia. Expert Rev Gastroenterol Hepatol. 2024;18:407–20. 39072626 10.1080/17474124.2024.2386367

[5] Hamashima C, Yoshimura K, Fukao A. A study protocol for expanding the screening interval of endoscopic screening for gastric cancer based on individual risks: prospective cohort study of gastric cancer screening. Ann Transl Med. 2020;8:1604. 33437803 10.21037/atm-20-5949 PMC7791261

[6] Iijima K, Watanabe K, Shimodaira Y, et al. A final report on the real impact of the COVID-19 pandemic on the diagnosis of gastrointestinal cancer in Akita Prefecture, Japan in 2022. Tohoku J Exp Med. 2024;263:161–8. 38658347 10.1620/tjem.2024.J025

[7] Asanuma K, Iijima K, Shimosegawa T. Gender difference in gastro-esophageal reflux diseases. World J Gastroenterol. 2016;22:1800–10. 26855539 10.3748/wjg.v22.i5.1800 PMC4724611

[8] Fukuda S, Watanabe K, Kubota D, et al. Cancer risk by length of Barrett’s esophagus in Japanese population: a nationwide multicenter retrospective cohort study. J Gastroenterol. 2024;59:887–95. 39150527 10.1007/s00535-024-02139-2

[9] Examination Committee of Criteria for “Obesity Disease” in Japan, Japan Society for the Study of Obesity. New criteria for “obesity disease” in Japan. Circ J. 2002;66:987–92. 12419927 10.1253/circj.66.987

[10] Kimura K, Takemoto T. An endoscopic recognition of the atrophic border and its significance in chronic gastritis. Endoscopy. 1969;3:87–97.

[11] Sugano K, Spechler SJ, El-Omar EM, et al. Kyoto international consensus report on anatomy, pathophysiology and clinical significance of the gastro-oesophageal junction. Gut. 2022;71:1488–514. 35725291 10.1136/gutjnl-2022-327281 PMC9279854

[12] Kitagawa Y, Ishihara R, Ishikawa H, et al. Esophageal cancer practice guidelines 20 edited by the Japan Esophageal society: part 2. Esophagus. 2023;20:373–89. 36995449 10.1007/s10388-023-00994-1 PMC10235142

[13] Kanda Y. Investigation of the freely available easy-to-use software ‘EZR’ for medical statistics. Bone Marrow Transplant. 2013;48:452–8. 23208313 10.1038/bmt.2012.244 PMC3590441

[14] van Blankenstein M, Looman CW, Johnston BJ, et al. Age and sex distribution of the prevalence of Barrett’s esophagus found in a primary referral endoscopy center. Am J Gastroenterol. 2005;100:568–76. 15743353 10.1111/j.1572-0241.2005.40187.x

[15] Ford AC, Forman D, Reynolds PD, et al. Ethnicity, gender, and socioeconomic status as risk factors for esophagitis and Barrett’s esophagus. Am J Epidemiol. 2005;162:454–60. 16076833 10.1093/aje/kwi218

[16] Matsuhashi N, Sakai E, Ohata K, et al. Surveillance of patients with long-segment Barrett’s esophagus: a multicenter prospective cohort study in Japan. J Gastroenterol Hepatol. 2017;32:409–14. 27416773 10.1111/jgh.13491

[17] Norita K, Koike T, Saito M, et al. Long-term endoscopic surveillance for Barrett’s esophagus in Japan: multicenter prospective cohort study. Dig Endosc. 2021;33:1085–92. 33277694 10.1111/den.13910

[18] Rodriguez S, Mattek N, Lieberman D, et al. Barrett’s esophagus on repeat endoscopy: should we look more than once? Am J Gastroenterol. 2008;103:1892–7. 18564120 10.1111/j.1572-0241.2008.01892.x PMC3922226

[19] Manabe N, Haruma K, Imamura H, et al. Does short-segment columnar-lined esophagus elongate during a mean follow-up period of 5.7 years? Dig Endosc. 2011;23:166–72. 21429023 10.1111/j.1443-1661.2010.01073.x

[20] Moawad FJ, Young PE, Gaddam S, et al. Barrett’s oesophagus length is established at the time of initial endoscopy and does not change over time: results from a large multicentre cohort. Gut. 2015;64:1874–80. 25652086 10.1136/gutjnl-2014-308552

[21] Qumseya B, Yang S, Guo Y. Trends in prevalence of esophageal adenocarcinoma: findings from a statewide database of over 6 million patients. Endosc Int Open. 2024;12:E218–26. 38362358 10.1055/a-2221-7974 PMC10869210

[22] Shiota S, Singh S, Anshasi A, et al. Prevalence of Barrett’s esophagus in Asian countries: a systematic review and meta-analysis. Clin Gastroenterol Hepatol. 2015;13:1907–18. 26260107 10.1016/j.cgh.2015.07.050 PMC4615528

[23] Shinkai H, Iijima K, Koike T, et al. Association between the body mass index and the risk of Barrett’s esophagus in Japan. Digestion. 2014;90:1–9. 25074386 10.1159/000357776

[24] Kubota D, Takahashi Y, Yamamichi N, et al. Analysis of Barrett’s esophagus and its risk factors: a cross-sectional study of 10,122 subjects at a Japanese health examination center. Digestion. 2022;103:411–20. 36075194 10.1159/000526154 PMC9808710

[25] Adachi K, Ishimura N, Kishi K, et al. Prevalence of Barrett’s epithelium shown by endoscopic observations with linked color imaging in subjects with different *H.**pylori* infection statuses. Intern Med. 2021;60:667–74. 32999237 10.2169/internalmedicine.5676-20 PMC7990643

[26] Kodama S, Watanabe K, Shimodaira Y, et al. Development of a prediction score for Barrett’s esophagus in Japanese health checkup settings. Esophagus. 2024;21:552–62. 39158677 10.1007/s10388-024-01079-3

[27] Matsuzaki J, Suzuki H, Asakura K, et al. Etiological difference between ultrashort- and short-segment Barrett’s esophagus. J Gastroenterol. 2011;46:332–8. 21132333 10.1007/s00535-010-0353-y

[28] Corley DA, Kubo A, Levin TR, et al. Abdominal obesity and body mass index as risk factors for Barrett’s esophagus. Gastroenterol. 2007;133:34–41. 10.1053/j.gastro.2007.04.046 17631128

[29] Singh S, Sharma AN, Murad MH, et al. Central adiposity is associated with increased risk of esophageal inflammation, metaplasia, and adenocarcinoma: a systematic review and meta-analysis. Clin Gastroenterol Hepatol. 2013;11:1399–412. 23707461 10.1016/j.cgh.2013.05.009 PMC3873801

[30] Watanabe K, Koizumi S, Shirane K, et al. Visceral obesity is associated with an increased risk of developing esophago-gastric junctional adenocarcinoma in Japan: a population-based case-control study in Akita Prefecture. Esophagus. 2022;19:477–85. 34993674 10.1007/s10388-021-00906-1

[31] Ishii K, Ogawa W, Kimura Y, et al. Diagnosis of sarcopenic obesity in Japan: Consensus statement of the Japanese Working Group on Sarcopenic Obesity. Geriatr Gerontol Int. 2024 (in press). 10.1111/ggi.14978 PMC11503547 39253949

[32] Wannamethee SG, Atkins JL. Muscle loss and obesity: the health implications of sarcopenia and sarcopenic obesity. Proc Nutr Soc. 2015;74:405–12. 25913270 10.1017/S002966511500169X

[33] Kim YM, Kim JH, Baik SJ, et al. Sarcopenia and sarcopenic obesity as novel risk factors for gastric carcinogenesis: a health checkup cohort study. Front Oncol. 2019;9:1249. 31799199 10.3389/fonc.2019.01249 PMC6868021

[34] Lear SA, Humphries KH, Kohli S, et al. The use of BMI and waist circumference as surrogates of body fat differs by ethnicity. Obes (Silver Spring). 2007;15:2817–24. 10.1038/oby.2007.334 18070773

[35] Lim U, Ernst T, Buchthal SD, et al. Asian women have greater abdominal and visceral adiposity than Caucasian women with similar body mass index. Nutr Diabetes. 2011;1: e6. 23449381 10.1038/nutd.2011.2 PMC3302135

[36] Abe Y, Ohara S, Koike T, et al. The prevalence of *Helicobacter**pylori* infection and the status of gastric acid secretion in patients with Barrett’s esophagus in Japan. Am J Gastroenterol. 2004;99:1213–21. 15233656 10.1111/j.1572-0241.2004.30313.x

