# Supplementary material for: Age distribution and risk factors for Barrett's esophagus by sex at health check-up settings in Japan
Source: J Gastroenterol. 2025 Feb 10;60(7):820–8. doi: 10.1007/s00535-025-02222-2 (PMC12176911; doi:10.1007/s00535-025-02222-2)
Supplement: Supplementary file 1 — Supplementary file1 (DOCX 23 KB) [file 535_2025_2222_MOESM1_ESM.docx]

**Supplemental Table 1: Regression analyses for factors associated with long-segment Barrett’s esophagus in men**

|  |  | Univariable | |  | Multivariable | |
| --- | --- | --- | --- | --- | --- | --- |
| Variables | Ref | OR (95% CI) | P values |  | AOR (95% CI) | P values |
| Age ≥65 years | <65 | 0.70 (0.32-1.55) | 0.38 |  | 0.86 (0.26-2.88) | 0.81 |
| BMI ≥25 kg/m^2^ | <25 | 0.83 (0.44-1.57) | 0.56 |  | 0.48 (0.22-1.04) | 0.063 |
| Central obesity* (Yes) | No | 2.15 (1.12-4.12) | 0.021 |  | 3.18 (1.54-6.53) | 0.0017 |
| Ever smokers | Never | 1.22 (0.63-2.33) | 0.56 |  | 1.05 (0.54-2.03) | 0.88 |
| Ever drinkers | Never | 2.57 (0.62-10.7) | 0.19 |  | 2.46 (0.59-10.3) | 0.21 |
| Gastric atrophy |  |  |  |  |  |  |
| Closed-type | None | 0.36 (0.17-0.76) | 0.007 |  | 0.38 (0.16-0.92) | 0.031 |
| Open-type | None | 0.18 (0.065-0.70) | 0.001 |  | 0.15 (0.036-0.65) | 0.011 |

*: Central obesity is defined as a waist circumference ≥85 cm for men and ≥90 cm for women.

AOR, adjusted odds ratio; BMI, body mass index; CI, confidence interval; OR, odds ratio

**Supplemental Table 2: Risks for Barrett’s esophagus (or long-segment Barrett’s esophagus) in men according to the 4 obesity categories**

| Obesity category | BE vs. control | | | | | |  | LSBE vs. control | | | | | |
| --- | --- | --- | --- | --- | --- | --- | --- | --- | --- | --- | --- | --- | --- |
|  |  | Univariable | |  | Multivariable* | |  |  | Univariable | |  | Multivariable* | |
|  | N (BE/control) | OR (95% CI) | P-value |  | AOR (95% CI) | P-value |  | N (LSBE/control) | OR (95% CI) | P-value |  | AOR (95% CI) | P-value |
| High BMI / Low WC | 47/167 | Ref. |  |  | Ref. |  |  | 0/167 | Ref. |  |  | Ref. |  |
| High BMI / High WC | 836/2,127 | 1.40 (1.00-1.95) | 0.049 |  | 1.38 (0.98-1.95) | 0.064 |  | 11/2,127 | n. a. | n. a. |  | n. a. | n. a. |
| Low BMI / Low WC | 1374/3,916 | 1.25 (0.88-1.73) | 0.19 |  | 1.27 (0.90-1.78) | 0.171 |  | 14/3,916 | n. a. | n. a. |  | n. a. | n. a. |
| Low BMI / High WC | 657/1,400 | 1.67 (1.19-2.33) | 0.003 |  | 1.73 (1.22-2.44) | 0.002 |  | 15/1,400 | n. a. | n. a. |  | n. a. | n. a. |

*: adjusted by age, smoking and drinking status, and gastric atrophy

AOR, adjusted odds ratio; BE, Barrette’s esophagus; BMI, body mass index; CI, confidence interval; LSBE, long-segment Barrett’s esophagus; OR, odds ratio; WC, waist circumference; n. a.: not available

**Supplemental Table 3: Regression analyses for factors associated with Reflux esophagitis in the entire cohort**

|  |  | Univariable | |  | Multivariable | |
| --- | --- | --- | --- | --- | --- | --- |
| Variables | Ref. | OR (95% CI) | P-value |  | AOR (95% CI) | P-value |
| Age ≥65 years | <65 | 0.77(0.70 – 0.85) | <0.001 |  | 1.17 (1.01-1.34) | 0.03 |
| Male Sex | female | 3.28 (3.01-3.58) | <0.001 |  | 3.21 (2.86-3.62) | <0.001 |
| BMI ≥25 kg/m^2^ | <25 | 1.97(1.82- 2.13) | <0.001 |  | 1.31 (1.17-1.47) | <0.001 |
| Central obesity* (Yes) | No | 2.44 (2.25-2.65) | <0.001 |  | 1.61 (1.44-1.80) | <0.001 |
| Ever smokers | Never | 1.36 (1.25-1.47) | <0.001 |  | 0.97 (0.89-1.06) | 0.54 |
| Ever drinkers | Never | 1.25 (1.13-1.37) | <0.001 |  | 0.88 (0.79-0.99) | 0.03 |
| Gastric atrophy |  |  |  |  |  |  |
| Closed-type | None | 0.63 (0.58-0.68) | <0.001 |  | 0.57 (0.51-0.63) | <0.001 |
| Open-type | None | 0.29 (0.26-0.33) | <0.001 |  | 0.25 (0.22-0.29) | <0.001 |

*: Central obesity is defined as a waist circumference ≥85 cm for men and ≥90 cm for women.

AOR, adjusted odds ratio; BMI, body mass index; CI, confidence interval; OR, odds ratio
